# Silymarin Activates c-AMP Phosphodiesterase and Stimulates Insulin Secretion in a Glucose-Dependent Manner in HIT-T15 Cells

**DOI:** 10.3390/antiox5040047

**Published:** 2016-12-12

**Authors:** Ran Meng, Jana Mahadevan, Elizabeth Oseid, Sara Vallerie, R. Paul Robertson

**Affiliations:** 1 Pacific Northwest Diabetes Research Institute, Seattle, WA 98122, USA; ranmengnju@gmail.com (R.M.); j.mahadevan@wustl.edu (J.M.); eoseid@gmail.com (E.O.); svallerie@uw.edu (S.V.); 2 Division of Metabolism, Endocrinology and Nutrition, Department of Medicine, University of Washington, Seattle, WA 98105, USA; 3 Department of Pharmacology, University of Washington, Seattle, WA 98105, USA

**Keywords:** insulin, phosphodiesterase, silymarin

## Abstract

Silymarin (SIL) is a flavonoid extracted from milk thistle seed that has been reported to decrease hyperglycemia in people with type 2 diabetes (T2D). However, it is not known whether SIL has direct secretory effects on β-cells. Using the β-cell line HIT-T15, SIL was shown to decrease intracellular peroxide levels and to augment glucose-stimulated insulin secretion (GSIS). However, the latter was observed using a concentration range of 25–100 µM, which was too low to affect endogenous peroxide levels. The stimulatory effect of SIL dissipated at higher concentrations (100–200 µM), and mild apoptosis was observed. The smaller concentrations of SIL also decreased cAMP phosphodiesterase activity in a Ca^2+^/calmodulin-dependent manner. The stimulatory effects of SIL on GSIS were inhibited by three different inhibitors of exocytosis, indicating that SIL’s mechanism of stimulating GSIS operated via closing β-cell K-ATP channels, and perhaps more distal sites of action involving calcium influx and G-proteins. We concluded that augmentation of GSIS by SIL can be observed at concentrations that also inhibit cAMP phosphodiesterase without concomitant lowering of intracellular peroxides.

## 1. Introduction

Type 2 diabetes (T2D) is primarily a polygenic disease that adversely affects function of islet β-cells, and one that is accentuated by obesity and insulin resistance. Chronic hyperglycemia causes the generation of reactive oxygen species (ROS) within islets, which in excess can lead to β-cell structural damage and dysfunction [1]. This knowledge has led to many studies examining the beneficial effects of antioxidants on hyperglycemia and abnormal insulin secretion in animal models of T2D (reviewed in [1]).

Silymarin (SIL) is a flavonoid extracted from milk thistle seed, and has been reported to decrease elevated levels of fasting glucose and hemoglobin A1c (HbA1c) in human T2D [2,3,4]. Silibin is a polyphenol that is the major antioxidant constituent of SIL. One of the known effects of SIL on non-islet cells is the inhibition of cyclic adenylmonophosphate (AMP) phosphodiesterase [5], an important regulator of beta cell function. However, no studies of the effects of SIL on glucose-stimulated insulin secretion (GSIS) have been reported previously, nor have other mechanisms involving the β-cell been clearly identified to explain the pharmacologic action(s) of SIL that decease elevated levels of fasting glucose and HbA1c in T2D.

Our studies were conducted using HIT-T15 cells (a glucose-responsive β-cell line) [6,7], and were designed to ascertain (1) whether SIL augments GSIS; (2) whether SIL secretory effects involve K-ATP channels and/or more distal sites in the exocytotic pathway; and (3) whether its effects on the β-cell are associated with alterations in cAMP-phosphodiesterase activity. Our approach was to establish SIL concentration–response curves for altering endogenous peroxide levels and GSIS. Dose–response curves for the effects on SIL on cAMP phosphodiesterase activity were also performed. Comparator studies for the efficacy of SIL to augment GSIS and inhibit cAMP phosphodiesterase were performed with three established cAMP phosphodiesterase inhibitors, namely, milrinone, rolipram, and 3-isobutyl-1-methylxanthine (IBMX).

## 2. Materials and Methods

### 2.1. Sources of Reagents

Silymarin and silibin were obtained from Sigma Aldrich, St. Louis, MO, USA; milrinone, rolipram, and IBMX were obtained from Cayman, Ann Arbor, MI, USA.

### 2.2. HIT-T15 Cells and Peroxide Levels

To determine the efficacy of SIL as an antioxidant, HIT-T15 cells (1 million/well in Krebs Ringer Buffer (KRB)) were studied in the presence of varying SIL concentrations (25–500 µM) in cultures containing 11.1 mM glucose. All passage numbers for HIT-T15 cells were between 72 and 79 to ensure responsiveness to glucose-stimulated insulin secretion [6,7]. Peroxide levels were measured from samples obtained after 2 h incubation according to the method of Sharma and Gupta [8]. Assessment of apoptosis was performed by fluorescent microscopy according to the terminal deoxynucleo transferase nick labeling (TUNEL) method [9].

### 2.3. HIT-T15 Cells and Insulin Secretion and Content

To determine the effects of SIL on β-cell function, one million HIT-T15 cells were incubated for 2 h in Rosewell Park Memorial Institute-1640 (RPMI-1640) media containing 11.1 mM glucose and varying concentrations of SIL (0–500 µM). The cells were then washed twice with KRB buffer, and harvested 2 h before static incubation for 2 h in 0.2 mM glucose to establish a basal, non-stimulated condition, and then for 2 more h in 0.2 or 11.1 mM glucose, as previously reported [6,7]. Insulin concentrations in the buffer were measured, and acid–alcohol extractions of the cells were determined at the conclusion of the GSIS studies to determine insulin content of the cells, as previously described [6,7]. To determine likely intracellular sites of action for SIL, three classic inhibitors of exocytosis (epinephrine (1 µM), nifedipine (20–50 µM), or diazoxide (50 µM)) were used during GSIS studies, as previously described [10].

### 2.4. HIT-T15 Cells and cAMP Phosphodiesterase Activity

To determine the effects of SIL on cAMP phosphodiesterase activity, hydrolysis of 1 µM cAMP in the presence increasing concentrations of SIL (0.1–100 µM) in the presence and absence of Ca^2+^/calmodulin were examined according to the method of Rybalkin et al. [11]. Comparator studies with SIL and other phosphodiesterase inhibitors for effects on GSIS were conducted with silibin, milrinone, rolipram, and IBMX.

### 2.5. Statistical Analysis

Statistical examination of the data was performed using non-paired comparisons by Students *t*-test or analysis of variance (ANOVA), where appropriate.

## 3. Results

### 3.1. Silymarin Effects on Intracellular Peroxide Levels

**Figure 1:**
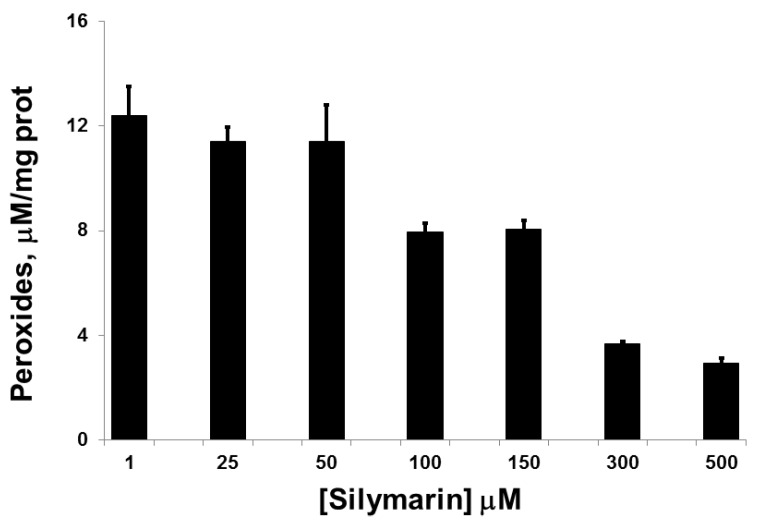
Levels of intracellular peroxides in HIT-T15 cells incubated in the presence of glucose (11.1 mM) and exposed to increasing concentrations of Silymarin (SIL). A drug concentration-related decrease in peroxides was observed (1.0 vs. 100 µM, *p* < 0.01, *n* = 3 experiments).

The threshold for the inhibitory effect of SIL on HIT-T15 intracellular peroxide levels was between 50–100 µM, with increasing inhibition observed from 100 to 500 µM (Figure 1).

### 3.2. Silymarin Effects on Insulin Secretion, Insulin Content, and Apoptosis

**Figure 2:**
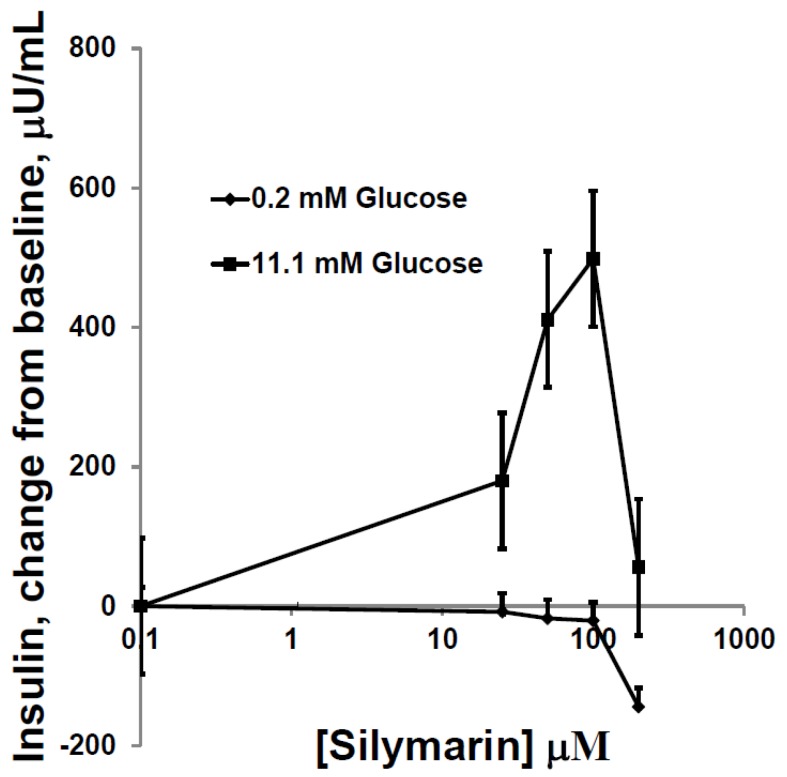
Insulin levels secreted by HIT-T15 cells in static incubations in buffer containing either 0.2 or 11.1 mM glucose for 2 h in the presence of increasing concentrations of SIL. No increase in insulin levels was observed using the non-stimulatory glucose concentration of 0.2 mM with or without inclusion of SIL in the incubate. However, in the presence of 11.1 mM glucose, an increase in baseline insulin was observed as well as augmentation of this increase by increasing concentrations of Silymarin, with a maximum effect 100 µM SIL (*p* < 0.01) and a decrease in response when using 200 µM. Data = mean ± SE; *n* = 8 experiments.

Incubation of cells with increasing concentrations of SIL in buffer containing 0.2 µM glucose had no effects on insulin secretion. However, when buffer contained 11.1 rather than 0.2 µM glucose, SIL augmented glucose-induced insulin secretion with maximal effect at 50–100 µM (Figure 2). SIL (25–500 µM) did not affect the insulin content of the cells in these triplicate experiments (range of values using 0 to 100 µM SIL = 21,000 ± 3000 vs. 19,823 ± 2682; µU/mg prot.; *p* = ns). With SIL concentrations higher than 100 µM, augmentation of glucose-dependent insulin secretion dissipated, and exposure to >100 µM SIL caused mild apoptosis (data not shown). Drug concentrations above 200 µM destroyed HIT-T15 cells, as evidenced by fragmented, floating cells in the culture plates.

**Figure 3:**
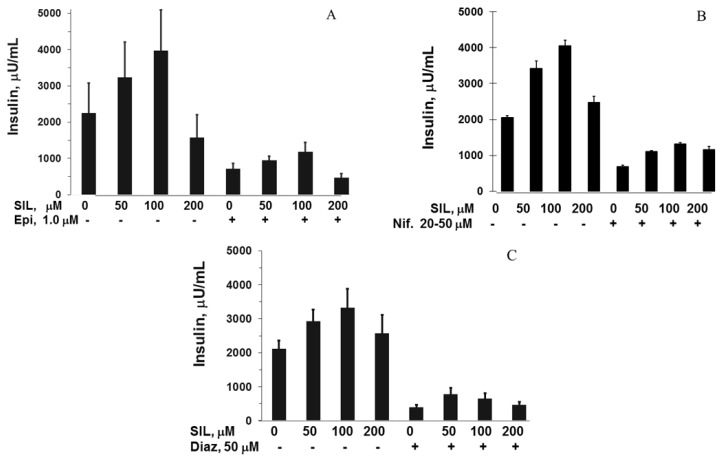
Insulin secretory responses from HIT-T15 cells in 2 h static incubations containing 11.1 mM glucose in increasing concentrations of SIL with and without three inhibitors of exocytosis. (**A**) Epinephrine (1 µM); (**B**) Nifedipine (20–50 µM); (**C**) Diazoxide (50 µM). In all experiments, SIL 50–100 µM augmented glucose-stimulated insulin secretion (GSIS), an effect that was maximum at 100 µM SIL. In all cases, this response was significantly decreased when the three inhibitors of exocytosis were present (all *p* = < 0.05–0.001). All experiments performed using three to five experiments. Data = mean ± SE.

To assess whether the stimulatory effects of SIL on GSIS were mediated by the beta cell exocytotic pathway, studies with three inhibitors of exocytosis were performed. Incubation with epinephrine (1 µM)—a distal G-protein-dependent inhibitor of exocytosis—inhibited both GSIS and SIL enhancement of GSIS (Figure 3A). Similarly, the calcium channel blocker nifedipine (20–50 µM), and diazoxide (50 µM), which maintains K-ATP channels in an open position and prevents depolarization, also inhibited both GSIS and SIL augmentation of GSIS (Figure 3B,C).

### 3.3. The Effect of SIL on cAMP Phosphodiesterase Activity and Comparison to other Inhibitors of cAMP Phosphodiestrerase Activity on GSIS and cAMP Phosphodiesterase Activity

**Figure 4:**
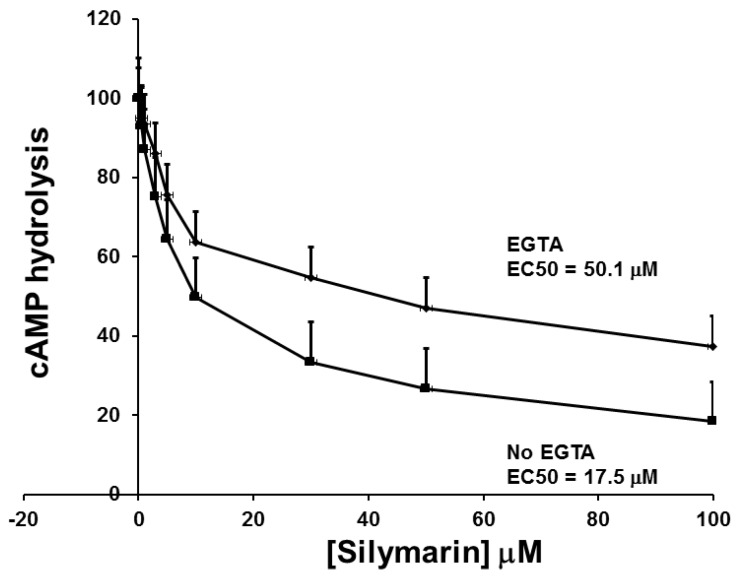
cAMP hydrolysis with increasing concentrations of SIL in the presence of EGTA (a specific chelator for Ca^2+^/calmodulin) and in its absence. A drug concentration-related decrease in cAMP hydrolysis was observed with differing EC_50_ values (No EGTA = 17.5 µM; EGTA = 50.1 µM). Data are mean ± SE; experiments performed with *n* = 3.

SIL decreased cAMP phosphodiesterase activity in a concentration-dependent manner that was attenuated by the presence of EGTA (a specific chelator for Ca^2+^/calmodulin; absence: EC_50_ (the concentration giving a half-maximal effect) = 6.7 ± 0.6 vs. presence: 26.0 ± 2.3 µM, mean ± SE; *p* < 0.001; Figure 4).

**Figure 5:**
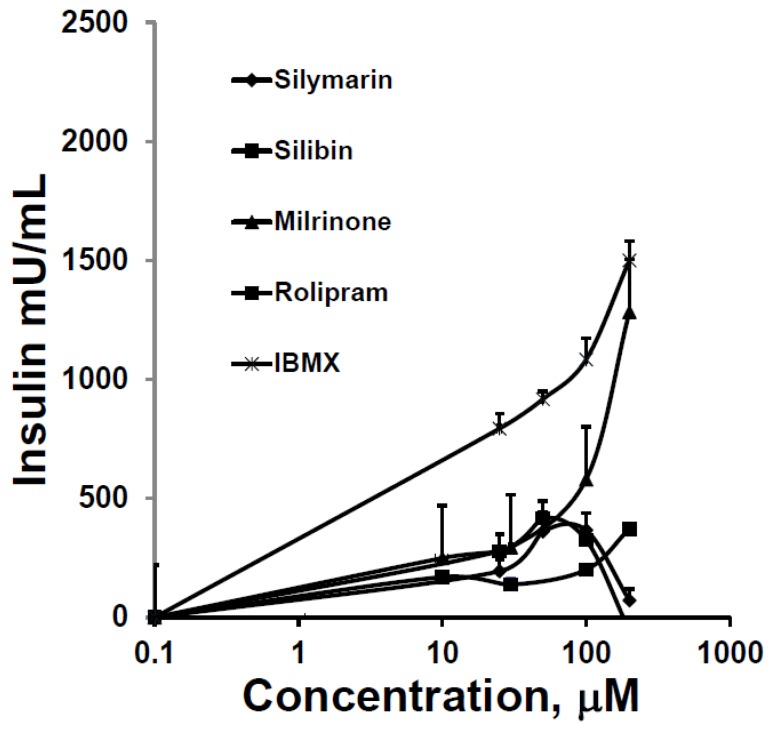
Augmentation of GSIS over baseline from HIT-T15 cells in static incubations containing 11.1 mM glucose over 2 h. A series of increasing concentrations of phosphodiesterase inhibitors was compared. The first evidence for the augmentation of GSIS occurred at a concentration of 20 µM for all drugs, and maximum responses occurred at 100 µM, except for milrinone and 3-isobutyl-1-methylxanthine (IBMX), whose augmentation effects continued to rise. Data = mean ± SE; experiments performed in triplicate.

SIL and silibin stimulatory effects on GSIS were quantitatively similar to each other, greater than that of rolipram, and substantially less than that of milrinone and IBMX (Figure 5).

## 4. Discussion

Mechanisms through which SIL might decrease levels of hyperglycemia and HbA1c in T2D [2,3,4] have not been previously reported. SIL in the concentration range of 100–500 µM had a suppressive effect on endogenous peroxide levels in HIT-T15 cells. SIL had no effect on β-cell function in cells exposed to non-stimulatory levels of glucose, but did enhance insulin responses to a stimulatory concentration of glucose. In this respect, SIL is similar to Glucagon-like peptide-1) GLP-1 but unlike other agents—such as sulphonylureas and glucagon—which stimulate β-cells regardless of ambient glucose concentrations. Importantly, the enhancement of GSIS was observed in a concentration range of 25–100 µM SIL, concentrations that had no effect on endogenous peroxide levels. Moreover, the greater concentrations of SIL (100–200 µM) that were required to decrease endogenous peroxide levels caused mild apoptosis of the cells, and greater concentrations caused cell death. No changes in insulin content were observed over a SIL concentration range of 0–100 µM, which argues against the possibility that SIL increased insulin levels in the culture media by damaging the cells and thereby causing insulin leakage. In this regard, all three inhibitors of exocytosis significantly diminished the augmentation of GSIS by SIL, clearly pointing toward a SIL effect at sites in the exocytotic pathway. The most proximally acting of these inhibitors was diazoxide, a drug that maintains K-ATP channels in the open position, thereby maintaining a polarized state and diminishing insulin exocytosis [10]. This suggests the mechanism of SIL’s action in enhancing GSIS involves the closure of K-ATP channels. The data from the experiments with the other two exocytotic inhibitors—epinephrine and nifedipine—are consistent with this conclusion, but allow for the possibility that sites distal to the K-ATP channels may also be affected by SIL. Most germane to the questions addressed by this study is that it is unlikely that the primary augmentation effect of SIL on insulin secretion is related to its antioxidant property, because its effect of decreasing intracellular peroxides required greater concentrations (100–500 µM) than those that augmented insulin secretion (25–100 mM). Higher SIL concentrations had a tendency to cause apoptosis, and the highest caused cell death.

SIL was first reported to have beneficial effects in controlling glycemia in T2D by Velussi et al. in 1997 [2]. They compared the effects of standard therapy versus standard therapy plus SIL (200 mg three times daily) in two groups of 30 subjects for one year. They observed significant decrements in fasting and mean daily glucose levels, daily glycosuria, glycosylated hemoglobin levels, fasting plasma insulin levels (but not glucose-stimulated insulin levels), and daily insulin requirements in the SIL-treated subjects. In 2006, Huseini et al. [4] reported results from a comparison of 200 mg SIL three times daily added or not added to a regimen of metformin and glibenclamide in two groups of 25 type T2D subjects using a randomized, placebo-controlled, double-blind design. After 4 months, the SIL-treated group had significant decrements in fasting glucose and a significant average drop in HbA1c of 1%. In 2007, Hussain [3] compared SIL (200 mg/day) vs. placebo added to glibenclamide (20 mg/day). After 3 months of treatment, SIL was reported to significantly lower fasting glucose by an average of 2 mM and HbA1c by an average of 1.4%. These authors also reported an average drop in body mass index of 2.9, which would have increased insulin sensitivity. None of these studies examined possible mechanisms of action of SIL in terms of oxidative stress or glucose-induced insulin secretion. In each case, the assumption was made that the beneficial effect of SIL on hyperglycemia was via its antioxidant effect. The reasonability of this premise is substantiated by our earlier studies that examined antioxidant drugs in β-cell lines, isolated islets, and animal studies in vivo [1,12,13]. In those studies, chronic hyperglycemia was associated with oxidative stress, and antioxidants were shown to ameliorate oxidative stress and improve β-cell responses to glucose (reviewed in [1]). Nonetheless, our current study suggests a novel alternative mechanism for SIL; i.e., augmentation of GSIS at exocytotic sites as early as K-ATP channels via inhibition of cAMP-phosphodiesterase.

## 5. Conclusions

These studies suggest that SIL has two potential mechanisms of action to improve β-cell function in T2D. One is the enhancement of GSIS via the closure of K-ATP channels, depolarization of β-cells, and enhanced exocytosis. The other is via the diminution of β-cell endogenous peroxides and oxidative stress. However, the EC_50_ values for SIL for decreasing cAMP phosphodiesterase activity and augmenting glucose-induced insulin secretion are in the 50–100 µM range, whereas the antioxidant effect of SIL requires a concentration of 100–150 µM. Our data suggest that the effect of low concentrations of SIL to augment GSIS is more closely related to the inhibition of cAMP phosphodiesterase than to its antioxidant effect.
